# SNP Markers and Their Impact on Plant Breeding

**DOI:** 10.1155/2012/728398

**Published:** 2012-12-18

**Authors:** Jafar Mammadov, Rajat Aggarwal, Ramesh Buyyarapu, Siva Kumpatla

**Affiliations:** ^1^Department of Trait Genetics and Technologies, Dow AgroSciences LLC, 9330 Zionsville Road, Indianapolis, IN 46268, USA; ^2^Department of Biotechnology Regulatory Sciences, Dow AgroSciences LLC, 9330 Zionsville Road, Indianapolis, IN 46268, USA

## Abstract

The use of molecular markers has revolutionized the pace and precision of plant genetic analysis which in turn facilitated the implementation of molecular breeding of crops. The last three decades have seen tremendous advances in the evolution of marker systems and the respective detection platforms. Markers based on single nucleotide polymorphisms (SNPs) have rapidly gained the center stage of molecular genetics during the recent years due to their abundance in the genomes and their amenability for high-throughput detection formats and platforms. Computational approaches dominate SNP discovery methods due to the ever-increasing sequence information in public databases; however, complex genomes pose special challenges in the identification of informative SNPs warranting alternative strategies in those crops. Many genotyping platforms and chemistries have become available making the use of SNPs even more attractive and efficient. This paper provides a review of historical and current efforts in the development, validation, and application of SNP markers in QTL/gene discovery and plant breeding by discussing key experimental strategies and cases exemplifying their impact.

## 1. Introduction

Allelic variations within a genome of the same species can be classified into three major groups that include differences in the number of tandem repeats at a particular locus [microsatellites, or simple sequence repeats (SSRs)] [1], segmental insertions/deletions (InDels) [2], and single nucleotide polymorphisms (SNPs) [3]. In order to detect and track these variations in the individuals of a progeny at DNA level, researchers have been developing and using genetic tools called molecular markers [4]. Although SSRs, InDels, and SNPs are the three major allelic variations discovered so far, a plethora of molecular markers were developed to detect the polymorphisms that resulted from these three types of variation [5]. Evolution of molecular markers has been primarily driven by the throughput and cost of detection method and the level of reproducibility [6]. Depending on detection method and throughput, all molecular markers can be divided into three major groups: (1) low-throughput, hybridization-based markers such as restriction fragment length polymorphisms (RFLPs) [4]; (2) medium-throughput, PCR-based markers that include random amplification of polymorphic DNA (RAPD) [7], amplified fragment length polymorphism (AFLP) [8], SSRs [9]; (3) high-throughput (HTP) sequence-based markers: SNPs [3]. In late eighties, RFLPs were the most popular molecular markers that were widely used in plant molecular genetics because they were reproducible and codominant [10]. However, the detection of RFLPs was an expensive, labor- and time-consuming process, which made these markers eventually obsolete. Moreover, RFLP markers were not amenable to automation. Invention of PCR technology and the application of this method for the rapid detection of polymorphisms overthrew low-throughput RFLP markers, and new generation of PCR-based markers emerged in the beginning of nineties. RAPD, AFLP, and SSR markers are the major PCR-based markers that research community has been using in various plant systems. RAPDs are able to simultaneously detect polymorphic loci in various regions of a genome [11]. However, they are anonymous and the level of their reproducibility is very low due to the non-specific binding of short, random primers. Although AFLPs are anonymous too, the level of their reproducibility and sensitivity is very high owing to the longer +1 and +3 selective primers and the presence of discriminatory nucleotides at 3′ end of each primer. That is why AFLP markers are still popular in molecular genetics research in crops with little to zero reference genome sequence available [12]. However, AFLP markers did not find widespread application in molecular breeding owing to the lengthy and laborious detection method, which was not amenable to automation either. Therefore, it was not surprising that soon after the discovery of SSR markers in the genome of a plant, they were declared as “markers of choice” [13], because SSRs were able to eliminate all drawbacks of the above-mentioned DNA marker technologies. SSRs were no longer anonymous; they were highly reproducible, highly polymorphic, and amenable to automation. Despite the cost of detection remaining high, SSR markers had pervaded all areas of plant molecular genetics and breeding in late 90s and the beginning of 21st century. However, during the last five years, the hegemony of medium-throughput SSRs was eventually broken by SNP markers. First discovered in human genome, SNPs proved to be universal as well as the most abundant forms of genetic variation among individuals of the same species [14]. Although SNPs are less polymorphic than SSR markers because of their biallelic nature, they easily compensate this drawback by being abundant, ubiquitous, and amenable to high- and ultra-high-throughput automation. However, despite these obvious advantages, there were only a limited number of examples of application of SNP markers in plant breeding by 2009 [15]. In this paper, we tried to summarize the recent progress in the utility of SNP markers in plant breeding.

## 2. SNP Discovery in Complex Plant Genomes

While SNP discovery in crops with simple genomes is a relatively straightforward process, complex genomes pose serious obstacles for the researchers interested in developing SNPs. One of the major problems is the highly repetitive nature of the plant genomes [16]. Prior to the emergence of next-generation sequencing (NGS) technologies, researchers used to rely on different experimental strategies to avoid repetitive portions of the genome. These include discovery of SNPs experimentally by resequencing of unigene-derived amplicons using Sanger's method [17] and *in silico* SNP discovery through the mining of SNPs within EST databases followed by PCR-based validation [18]. Although these approaches allowed the detection of gene-based SNPs, their frequency is generally low in conserved genic regions, and they were unable to discover SNPs located in low-copy noncoding regions and intergenic spaces. Additionally, amplicon resequencing was an expensive and labor-intensive procedure [15]. As many crops are ancient tetraploids with mosaics of scattered duplicated regions [19], *in silico* and experimental mining of EST databases resulted in the discovery of a large number of nonallelic SNPs that represented paralogous sequences and were suboptimal for application in molecular breeding [20]. Recent emergence of NGS technologies such as 454 Life Sciences (Roche Applied Science, Indianapolis, IN), HiSeq (Illumina, San Diego, CA), SOLiD and Ion Torrent (Life Technologies Corporation, Carlsbad, CA) has eliminated the problems associated with low throughput and high cost of SNP discovery [21]. Transcriptome resequencing using NGS technologies allows rapid and inexpensive SNP discovery within genes and avoids highly repetitive regions of a genome [22]. This methodology was successfully applied in several plant genomes, including maize [23], canola [24], eucalyptus [25], sugarcane [26], tree species [27], wheat [28], avocado [29], and black currant [30]. Originally developed for human disease diagnostic research, the NimbleGen sequence capture technology (Roche Applied Science, IN) [31] brought the detection of gene-based SNPs in plants into higher throughput and coverage level [32]. This technology consists of exon sequence capture and enrichment by microarray followed by NGS for targeted resequencing. Similar in-solution target capture technologies, such as Agilent SureSelect, are also commercially available for genome/exome mining studies. However, this technology would be efficient only for crops with available reference genome sequence or large transcriptome (EST) datasets, since the design of capture probes requires these reference resources.

Despite the attractiveness of SNP discovery via transcriptome or exome resequencing, this process is targeted, focusing solely on coding regions. It is obvious that the availability of SNPs within coding sequences is a very powerful tool for molecular geneticists to detect a causative mutation [33]. However, often QTL are located in noncoding regulatory sequences such as enhancers or locus control regions, which could be located several megabases away from genes within intergenic spaces [34]. Discovery of SNPs located within those regulatory elements via transcriptome or exon sequencing is limited. In order to discover SNPs in a genome-wide fashion and avoid repetitive and duplicated DNA, it is very important to employ genome complexity reduction techniques coupled with NGS technologies. Several genome complexity reduction techniques have been developed over the years, including High Cot selection [35], methylation filtering [36], and microarray-based genomic selection [37]. These techniques mainly reduce the number of repetitive sequences but lack the power to recognize and eliminate duplicated sequences, which cause the detection of false-positive SNPs. Unlike the above-mentioned techniques, recently developed genome complexity reduction technologies such as Complexity Reduction of Polymorphic Sequences (CRoPS) (Keygene N.V., Wageningen, The Netherlands) [38] and Restriction Site Associated DNA (RAD) (Floragenics, Eugene, OR, USA) [39] are computationally well equipped and capable of filtering out duplicated SNPs. These systems were successfully applied to discover SNPs in crops with [40] and without reference genome sequences [41].

Although several complexity reduction approaches are being developed to generate data from NGS platforms, it is often challenging to identify candidate SNPs in polyploid crops species such as potato, tobacco, cotton, canola, and wheat. In general, minor allele frequency could be used as a measure to identify candidate SNPs in diploid species [42]. However, in polyploid crops, you often find loci that are polymorphic within a single genotype due to the presence of either homoeologous loci from the individual subgenomes (homoeologous SNPs) or paralogous loci from duplicated regions of the genome. Such false positive SNPs are not useful for genetic mapping purposes and often lead to a lower validation rate during assays. Successful SNP validation in allopolyploids depends upon differentiation of the sequence variation classes [43]. Use of haplotype information beside the allelic frequency would help to identify homologous SNPs (true SNPs) from those of homoeologous loci (false positives). Bioinformatic programs such as HaploSNPer [44] would facilitate identification of candidate loci for assay design purposes in polyploid crops. Elimination of homoeologous loci for the assay design process would improve the validation rate. Such approaches could also be extended to other complex and highly repetitive diploid genomes such as barley. Complexity reduction approaches, combined with sophisticated computational tools, would expedite SNP discovery and validation efforts in polyploids.

Although CRoPS and RAD technologies are powerful tools to detect SNPs in genome-wide fashion, they can hardly be called HTP, because on an average only ~1,000 SNPs pass stringent quality control [40]. While these numbers are enough to generate genetic linkage maps of reasonable saturation and carry out preliminary QTL mapping, they are not adequate to implement genome-wide association studies (GWAS). Depending on the rate of linkage disequilibrium decay, GWAS might require several million genetic landmarks. From this point of view, genotyping-by-sequencing (GBS) technique offers many more opportunities. Discovery of a large number of SNPs using GBS was demonstrated in maize [45] and sorghum [46]. GBS not only increases the sequencing throughput by several orders of magnitude but also has multiplexing capabilities [47]. To eliminate a large portion of repetitive sequences, a type II restriction endonuclease, *Ape*KI, is applied to digest DNA prior to sequencing to generate reduced representation libraries (genome complexity reduction component), which are further subject to sequencing [47]. In polyploid crops, GBS might be challenging, but the associated complexity reduction methods could be used for SNP discovery. For discovery purposes, the availability of a reference genome is not an absolute requirement to implement GBS approach. However, in organisms that do not have a reference genome, GBS-derived SNPs must be validated using one of the techniques that are described in the following section, which might dramatically increase per marker price. Validation needs to be done primarily to discard paralogous SNPs. For organisms with a reference genome sequence, the validation step is replaced by *in silico* mapping of the sequenced fragments to the genome. Although GBS has the potential to discover several million SNPs, one of the major drawbacks of this technique is large numbers of missing data. To solve this problem, computational biologists developed data imputation models such as BEAGLE v3.0.2 [48] and IMPUTE v2 [49], to bring imputed data as close as possible to the real data [50, 51].

## 3. SNP Validation and Modern Genotyping Platforms and Chemistries

The availability of reference sequence and sophisticated software does not always guarantee that the discovered SNP can be converted into a valid marker. In order to insure that the discovered SNP is a Mendelian locus, it has to be validated. The validation of a marker is the process of designing an assay based on the discovered polymorphism and then genotyping a panel of diverse germplasm and segregating population. Compared to the collection of unrelated lines, a segregating population is more informative as a validation panel because it allows the inspection of the discriminatory ability and segregation patterns of a marker which helps the researcher to understand whether it is a Mendelian locus or a duplicated/repetitive sequence that escaped the software filter [40].

The most popular HTP assays/chemistries and genotyping platforms that are currently being used for SNP validation are Illumina's BeadArray technology-based Golden Gate (GG) [52] and Infinium assays [53], Life Technologies' TaqMan [54] assay coupled with OpenArray platform (TaqMan OpenArray Genotyping system, Product bulletin), and KBiosciences' Competitive Allele Specific PCR (KASPar) combined with the SNP Line platform (SNP Line XL; http://www.kbioscience.co.uk). These modern genotyping assays and platforms differ from each other in their chemistry, cost, and throughput of samples to genotype and number of SNPs to validate. The choice of chemistry and genotyping platform depends on many factors that include the length of SNP context sequence, overall number of SNPs to genotype, and finally the funds available to the researcher, because most of these chemistries still remain cost intensive. Comparative analyses of these four genotyping assays and platforms were described in Kumpatla et al. [55].

**Figure 1:**
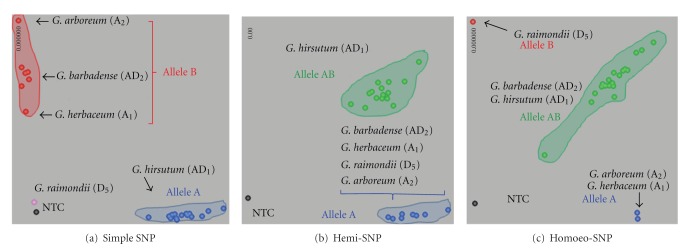
Segregation patterns of simple, hemi-, and homoeo-SNPs assayed using KASPar chemistry across tetraploid cotton species [*G*. *hirsutum* (AD_1_) and *G*. *barbadense* (AD_2_)] and their diploid progenitors [*G*. *arboreum*, *G*. *herbaceum* (A subgenome), and *G*. *raimondii* (D-subgenome)]. (a) Simple, or true SNP detects allelic variation between homologous loci of A subgenome of *G*. *hirsutum* (AD_1_) and *G*. *barbadense* (AD_2_). (b) Hemi-SNPs detect allelic variation in homozygous state in *G*. *barbadense* (AD_2_) and heterozygous state in *G*. *hirsutum* (AD_1_). (c) Homoeo-SNP detects homoelogous and/or paralogous loci both in A and D subgenomes, which are monomorphic between *G*. *barbadense* (AD_2_) and *G*. *hirsutum* (AD_1_). Allele calls depicted in blue, red, green, pink, and black represent alleles A, B, and AB, no amplification or missing locus, and no template control (NTC), respectively.

Though all genotyping chemistries and platforms are applicable to generate genotypic data in polyploid crops, analysis of SNP calls is somewhat challenging in polyploids due to multiallele combinations in the genotypes. SNPs in polyploid species can be broadly classified as simple SNPs, hemi-SNPs, and homoeo-SNPs. Here, we describe simple, hemi-, and homoeo-SNPs using an example of allele calls in tetraploid and diploid cotton species (Figure 1). Genomes of tetraploid cotton species, *Gossypium hirsutum* (AD_1_) and *G*. *barbadense* (AD_2_), consist of two subgenomes A and D, where A genome was derived from diploid progenitors, such as *G*. *herbaceum* (A_1_) and *G*. *arboreum* (A_2_), and D genome resulted from another diploid progenitor *G*. *raimondii* (D_5_). Simple, or true SNPs are markers that detect allelic variation between homologous loci of the same subgenome of two tetraploid samples. For example, in Figure 1(a), a SNP marker clearly detects polymorphism within A subgenomes of *G*. *hirsutum* (AD_1_) and *G*. *barbadense* (AD_2_) and separates samples into homozygous A (blue) and B (red) clusters. This marker does not discriminate polymorphism in D subgenome, because the D genome allele is absent there (pink dot in *G*. *raimondii*). In contrast to simple SNPs, hemi-SNPs detect allelic variation in the homozygous state in one sample and the heterozygous state in the other sample. In Figure 1(b), SNP marker detects both alleles (A and B) in *G*. *hirsutum* (heterozygous green cluster) and one allele A in *G*. *barbadense* (a homozygous blue cluster) and could be *vice versa*. Homoeo-SNPs detect homoeologous and possibly paralogous loci both in A and D subgenomes and result in monomorphic loci in tetraploid species (right image). In Figure 1(c) A genome progenitors (*G*. *herbaceum and G*. *arboreum*) had allele A (blue) and D genome progenitor (*G*. *raimondii*) had allele B (red), but both tetraploid species (*G*. *hirsutum and G*. *barbadense*) were grouped into heterozygous AB (green) cluster. As homoeo-SNPs can detect paralogous loci, the diploid progenitors both have different alleles.

Simple SNPs as well as hemi-SNPs are useful markers for genetic mapping and diversity screening studies. Simple SNPs segregate like the markers in diploids in most of the mapping populations and would account for approximately 10–30% of total polymorphic SNPs in various polyploid crop species. Hemi-SNPs form a major category (30–60%) of polymorphic SNPs in a polyploid crop species and could be used for genetic mapping purposes in F_2_, RIL, and DH populations. Homoeo-SNPs are of lesser value for mapping purposes as most of the genotypes result in heterologous loci due to polymorphism between the homoeologous genomes or duplicated loci within each of the polyploid genotypes [56].

## 4. Application of SNP Markers in Gene/QTL Discovery

### 4.1. Biparental Approach

Genetic mapping studies involve genetic linkage analysis, which is based on the concept of genetic recombination during meiosis [57]. This encompasses developing genetic linkage maps following genotyping of individuals in segregating populations with DNA markers covering the genome of that organism. Since their discovery in the 1980s, DNA-based markers have been widely used in developing saturated genetic linkage maps as well as for the mapping and discovery of genes/QTL. With the large-scale availability of the sequence information and development of HTP technologies for SNP genotyping, SNP markers have been increasingly used for QTL mapping studies. This is primarily, because SNPs are highly abundant in the genomes and, therefore, they can provide the highest map resolution compared to other marker systems [58, 59]. A review of the selected examples of QTL and gene discovery using SNP markers is presented below.

#### 4.1.1. Examples in Rice

A recent study on QTL analysis in rice for yield and three-yield-component traits, number of tillers per plant, number of grains per panicle, and grain weight compared a SNP-based map to that of a previous RFLP/SSR-based QTL map generated using the same mapping population [42]. Using the ultra-high-density SNP map, the authors showed that this map had more power and resolution relative to the RFLP/SSR map. This was clearly evident by the analysis of the two main QTL for grain weight, *kgw3a* (*GS3*) and *kgw5* (*GW5/qSW5*). Using the SNP bin map, *GW5/qSW5* QTL for grain width was accurately narrowed down to a 123 kb region as compared to the 12.4 Mb region based on the RFLP/SSR genetic map. Likewise, *GS3* QTL for grain length was mapped to a 197 kb interval in comparison to 6 Mb region with the RFLP/SSR genetic map. Beside the power and the resolution, maps based on high-density SNP markers are also highly suitable for fine mapping and cloning of QTL and at times SNPs on these maps are also functionally associated with the natural variation in the trait. In another QTL mapping project, SNP and InDel markers were used to fine map *qSH1* gene, a major QTL of seed shattering trait in rice [60]. The QTL were initially detected using RFLP and RAPD markers on F2 plants. Using large BC_4_F_2_ and BC_3_F_2_ populations in fine mapping approach with SNP and InDel markers, the authors mapped the functional natural variation to a 612 bp interval between the QTL flanking markers and discovered only one SNP. They further showed that this SNP in the 5′ regulatory region of the *qSH1* gene caused loss of seed shattering. Fine mapping approach was also taken to positionally clone the rice bacterial blight resistance gene *xa5*, by isolating the recombination breakpoints to a pair of SNPs followed by sequencing of the corresponding 5 kb region [61]. Several studies have shown that the SNPs and InDels are highly abundant and present throughout the genome in various species including plants [62–64]. SNP genotyping is a valuable tool for gene mapping, map-based cloning, and marker assisted selection (MAS) in crops [65]. A study was conducted to assess the feasibility of SNPs and InDels as DNA markers in genetic analysis and marker-assisted breeding in rice by analyzing these sequence polymorphisms in the genomic region containing *Piz* and *Piz-t* rice blast resistance genes and developing PCR-based SNP markers [65]. The authors discovered that SNPs were abundant in the *Piz* and *Piz-t* (averaging one SNP every 248 bp), while InDels were much lower. This dense distribution of SNPs helped in developing SNP markers in the vicinity of these genes. Advancements in rice genomics have led to mapping and cloning of several genes and QTL controlling agronomically important traits, enabled routine use of SNP markers for MAS, gene pyramiding, and marker-assisted breeding (MAB) [66–68].

#### 4.1.2. Examples in Maize

SNP markers have facilitated the dissection of complex traits such as flowering time in maize. Using a set of 5000 RILs, which represent the nested association mapping (NAM) population and genotyping with 1,200 SNP markers, the authors discovered that the genetic architecture of flowering time is controlled by small additive QTL rather than a single large-effect QTL [69]. The same NAM population was used for mapping resistance to northern leaf blight disease [70]. Twenty-nine QTL were discovered and candidate genes were identified with genome-wide NAM approach using 1.6 million SNPs. Proprietary SNP markers developed by companies are being predominantly used in their private breeding programs. A study from Pioneer Hi-Bred International Inc. reported identifying a high-oil QTL (*qHO6*) affecting maize seed oil and oleic acid contents. This QTL encodes an acyl-CoA:diacylglycerol acyltransferase (*DGAT1-2*), which catalyzes the final step of oil synthesis [71].

#### 4.1.3. Examples in Wheat

Recent advances in wheat genomics have led to the implementation of high-density SNP genotyping in wheat [72–75]. Gene-based SNP markers were developed for *Lr34/Yr18/Pm38* locus that confers resistance to leaf rust, stripe rust, and powdery mildew diseases [76]. These markers serve as efficient tools for MAS and MAB of disease resistant wheat lines. Another economically important wheat disease, Fusarium head blight (FHB), has been extensively studied. Several QTL controlling FHB resistance have been identified, with the most important being *Fhb1* [77]. Recently, SNP markers were mapped between the known flanking markers for *Fhb1* [78]. These new markers would be useful for MAS and fine mapping towards cloning the *Fhb1* gene. MAS in wheat has been extensively applied for simple traits that are difficult to score [79]. 

#### 4.1.4. Examples in Soybean

In order to improve the effectiveness of MAS and clone soybean aphid resistance gene, *Rag1*, fine mapping was done to accurately position the gene, which was previously mapped to a 12 cM interval [80]. The authors mapped the gene between two SNP markers that corresponded to a physical distance of 115 kb and identified several candidate genes. Similarly, another aphid resistance gene, *Rag2*, originally mapped to a 10 cM interval, was fine mapped to a 54 kb interval using SNP markers that were developed by resequencing of target intervals and sequence-tagged sites [81]. In another study that used a similar approach, the authors identified SNP markers tightly linked to a QTL conferring resistance to southern root-knot nematode by developing these SNP markers from the bacterial artificial chromosome (BAC) ends and SSR-containing genomic DNA clones [82]. In all of these examples the main idea behind the identification of closely linked SNP markers was to enhance the efficiency and cost effectiveness through MAS and increase the resolution within the target locus.

#### 4.1.5. Examples in Other Crops

In a study conducted in canola to map the *fad2* and *fad3* gene, single nucleotide mutations were identified by sequencing the genomic clones of these genes and subsequently SNP markers were developed [83]. Allele-specific PCR assays were developed to enable direct selection of desirable *fad2* and *fad3* alleles in marker-assisted trait introgression and breeding. In barley, SNP markers were identified that were linked to a covered smut resistance gene, *Ruh.7H*, by using high-resolution melting (HRM) technique [84]. In sugar beet, an anchored linkage map based on AFLP, SNP, and RAPD markers was developed to map QTL for *Beet necrotic yellow vein virus* resistance genes, *Rz4* [85] and *Rz5* [86]. A consensus genetic map based on EST-derived SNPs was developed for cowpea that would be an important resource for genomic and QTL mapping studies in this crop [87]. In one of the post-genomic era studies in 2002, the fine mapping and map-based cloning approaches were used to clone the *VTC2* gene in Arabidopsis [88]. The authors fine mapped the gene interval from ~980 kb region to a 20 kb interval with SNP and InDel markers. Additional nine candidate genes were identified in that interval and subsequently the underlying mutation was discovered. Although only a few examples that demonstrate the application of SNP markers in QTL mapping and genomic studies have been mentioned here, several other studies have been published in this area. Recent advances in HTP genotyping technologies and sequence information will further pave the way for rapid identification of causative variations and cloning of QTL of interest for use in MAB.

### 4.2. Genome-Wide Association Study Approach

GWAS is increasingly becoming a popular tool for dissecting complex traits in plants [89–92]. The idea behind GWAS is to genotype a large number of markers distributed across the genome so that the phenotype or the functional alleles will be in LD with one or few markers that could then be used in the breeding program. However, due to limited extent of LD, a greater number of markers are required for sufficient power to detect linkage between the marker and the underlying phenotypic variation. Several studies on association mapping in plants have been published and reviewed in the past [89, 90, 92, 93]. A few selected examples on the GWAS and candidate gene association (CGA) studies that utilized SNP markers are described below.

The successful use and first time demonstration of the power of GWAS was through the identification of a putative gene associated with a QTL in maize [94]. In that study, a single locus with major effect on oleic acid was mapped to a 4 cM genetic interval by using SNP haplotypes at 8,590 loci. The authors identified a fatty acid desaturase gene, *fad2*, at ~2 kb from one of the associated markers, and this was considered a likely causative gene. With the discovery of millions of SNPs in maize and the availability of tools such as NAM populations, GWAS was effectively applied to dissect the genetic architecture of leaf traits and it was also shown that variations at the *liguleless* genes contributed to more upright leaf phenotype [95]. Utility of the GWAS approach was demonstrated in barley through the mapping of a QTL for spot blotch disease resistance [96]. Using the diversity array technology (DArT) and SNP markers, the authors identified several QTL, some of which were not identified for this trait earlier. Another variant of the association mapping method is the CGA, where the association between one or few gene candidate loci and the trait of interest is tested. Using this approach 24 gene candidates were analyzed for association with the field resistance to late blight disease in potato and plant maturity. Nine SNPs were identified to be associated with maturity corrected resistance, explaining 50% of the genetic variance of this trait [97]. Two SNPs at the *allene oxide synthase 2* (*StAOS2*) gene locus were associated with the largest effect on the trait of interest. A GWAS approach was also successfully applied to understand the genetic architecture of complex diseases such as northern and southern corn leaf blights [70, 98]. Although the number of papers dedicated to the application of GWAS to reveal the genetic basis of agronomic traits is growing, the practical utility of minor QTL in molecular breeding is yet to be shown. As GWAS requires large number of molecular markers, the utility of GWAS in dissection of molecular basis of traits in polyploid crops such as canola, wheat, and cotton has been fairly limited due to the insufficient number of polymorphic markers and the absence of reference genome. However, recently developed associative transcriptomics method has a potential to overcome the above-mentioned shortages [99]. Harper et al. [99] leveraged differentially expressed transcriptome sequences to develop molecular markers in tetraploid crop *Brassica napus* and associated them with glucosinolate content variation in seeds. Due to the precision of this method, scientists were able to correlate specific deletions in canola genome with two QTL controlling the trait. Annotation of deleted regions revealed the orthologs of the transcription factor HAG1, which controlled aliphatic glucosinolate biosynthesis in *A*. *thaliana*. This research work gives an optimism on successful application of GWAS in polyploid crops.

## 5. Implementation of SNP Markers in Plant Breeding

Due to the availability of HTP SNP detection and validation technologies, the development of SNP markers becomes a routine process, especially in crops with reference genome. How has that influenced the application of SNP markers in plant breeding? In a review article, Xu and Crouch [100] indicated fairly low number of articles dedicated to the marker assisted selection for the 1986–2005 period. The combination of three key phrases (“marker-assisted selection” AND “SNP” AND “plant breeding”), indeed, shows only 637 articles at Google Scholar for that period. However, similar search for the period, spanning 2006 through 2012, demonstrates almost sevenfold (~4,560) increase in the number of articles indicating the application of SNPs in MAS. A vast majority of those publications are from public sector and primarily describe mapping QTL using SNPs and state the potential usefulness of those markers in MAS without any experimental support for that. For most of those research studies, QTL mapping is the final destination and further application of those markers in actual MAS leading to the development of varieties seldom happens. Fairly low impact of academic research in the MAS-based variety development can be explained by the lack of funding to complete the entire marker development pipeline (MDP), which can be long term and cost intensive. MDP includes several steps such as (1) population development, (2) initial QTL mapping, (3) QTL validation (testing in several locations and years and implementing fine mapping), and (4) marker validation (development of inexpensive but HTP and automation amenable assays) [101]. Every step of the development of markers linked to QTL is associated with numerous constraints, which may take several years and substantial funding to resolve. However, since 2006, there have been a few success stories about the development of varieties using SNPs in publications derived from academic research, including the development of submergence-tolerant rice cultivars [102], rice cultivars with improved eating, cooking, and sensory quality [103], leaf rust resistant wheat variety “Patwin” [104], and maize cultivar with low phytic acid [105]. Although the private sector does not normally release details of its breeding methodologies to the public, several papers published by Monsanto [106, 107], Pioneer Hi-bred [71], Syngenta [108], and Dow AgroSciences [109] indicate that commercial organizations are the main drivers in the application of SNP markers in MAS [110].

Current MAS strategies fit the breeding programs for the traits that are highly heritable and governed by a single gene or one major QTL that explains a large portion of the phenotypic variability. In reality, most of the agronomic traits such as yield, drought and heat tolerance, nitrogen and water use efficiency, and fiber quality in cotton have complex inheritance that is controlled by multiple QTL with minor effect. Use of one of those minor QTL in MAS will be inefficient because of its negligible effect on phenotype.

The MAS scheme using paternity testing has recently been proposed to address challenges associated with selection gains that can be achieved in outbred forage crops [111]. Paternity testing, a nonlinkage-based MAS scheme, improves selection gains by increasing parental control in the selection gain equation. The authors demonstrated paternity testing MAS in three red clover breeding populations by using permutation-based truncation selection for a biomass-persistence index trait and achieved paternity-based selection gains that were greater than double the selection gains based on maternity alone. The paternity was determined by using a small set (11) of SSR markers. SNP markers can also be used for paternity testing, but one would require a relatively larger number of SNP loci [112].

Meuwissen et al. [113] described a new methodology in plant breeding called genomic selection (GS) that was intended to solve problems related to MAS of complex traits. This methodology also applies molecular markers but in a different fashion in both diploid and polyploid crop species. Unlike MAS, in GS markers are not used for tracking a trait. In GS high-density marker coverage is needed to potentially have all QTL in LD with at least one marker. Then the comprehensive information on all possible loci, haplotypes, and marker effects across the entire genome is used to calculate genomic estimated breeding value (GEBV) of a particular line in the breeding population.

GS of superior lines can be carried out within any breeding population. In order to enable successful GS, the experimental population must be identified. The population should not be necessarily derived from bi-parental cross but must be representative of selection candidates in the breeding program to which GS will be applied [114]. The experimental population must be genotyped with a large number of markers. Taking into account the low cost of sequencing, the best choice is the GBS implementation, which will yield maximum number of polymorphisms. The sequence of the two events, that is, phenotypic and genotypic data collection, is arbitrary and can be done in parallel. When both phenotypic and genotypic data are ready, one can start “training” molecular markers [115]. In order to train the GS model, the effect of each marker is calculated computationally. The effect of a marker is represented by a number with a positive or negative sign that indicates the positive or negative effect, respectively, of a particular locus to the phenotype. When the effects of all markers are known, they are considered “trained” and ready to assess any breeding population different from the experimental one for the same trait. Availability of trained GS model does not require the collection of phenotypic data from new breeding populations. The same set of “trained” markers will be used to genotype a new breeding population. Based on genotypic data, the known effects of each marker will be summed and GEBV of each line will be calculated. The higher the GEBV value of an individual line, the more the chances that this line will be selected and advanced in the breeding cycle. Thus, GS using high-density marker coverage has a potential to capture QTL with major and minor effects and eliminate the need to collect phenotypic data in all breeding cycles. Also, the application of GS was demonstrated to reduce the number of breeding cycles and increase the annual gain [114]. One of the problems of GS is the level of GEBV accuracy. Simulation studies based on simulated and empirical data demonstrated that GEBV accuracy could be within 0.62–0.85. Heffner et al. [114] used previously reported GEBV accuracy of 0.53 and reported three- and twofold annual gain in maize and winter barley, respectively. The obvious advantages of GS over traditional MAS have been successfully proven in animal breeding [116]. Rapid evolution of sequencing technologies and HTP SNP genotyping systems are enabling generation and validation of millions of markers, giving a “cautious optimism” for successful application of GS in breeding for complex traits [117–120].

## 6. Conclusion

SNP markers have become extremely popular in plant molecular genetics due to their genome-wide abundance and amenability for high- to ultra-high-throughput detection platforms. Unlike earlier marker systems, SNPs made it possible to create saturated, if not, supersaturated genetic maps, thereby enabling genome-wide tracking, fine mapping of target regions, rapid association of markers with a trait, and accelerated cloning of gene/QTL of interest. On the flip side, there are some challenges that need to be addressed or overcome while using SNPs. For example, the biallelic nature of SNPs needs to be compensated by discovering and using a larger number of SNPs to arrive at the same or higher power as that of earlier-generation molecular markers. This could be cost prohibitive depending on the crop and the sequence resources available for that genome. Working with polyploid crops is another challenge where useful SNPs are only a small percentage of the total available polymorphisms. Creative strategies need to be employed to generate a reasonable number of SNPs in those species. The use of SNP markers in MAB programs has been growing at a faster pace and so is the development of technologies and platforms for the discovery and HTP screening of SNPs in many crops. SNP chips are currently available for several crops; however, one disadvantage is that these readily available chips are made based on SNPs discovered from certain genotypes and, therefore, may not be ideal for projects utilizing unrelated genotypes. This necessitates creation of multiple chips or the usage of technologies that permit design flexibility but are economical. Although GBS creates great opportunities to discover a large number of SNPs at lower per sample cost within the genotypes of interest, the lack of adequate computational capabilities such as reliable data imputation algorithms and powerful computers allowing quick processing and the storage of a large amount of sequencing data becomes a major bottleneck. Despite certain disadvantages or challenges, it is clear that SNP markers, in combination with genomics and other next-generation technologies, have been accelerating the pace and gains of plant breeding.
